# Optimization of the Red Tilapia (*Oreochromis* spp.) Viscera Hydrolysis for Obtaining Iron-Binding Peptides and Evaluation of In Vitro Iron Bioavailability

**DOI:** 10.3390/foods9070883

**Published:** 2020-07-06

**Authors:** Leidy J. Gómez, Nathalia A. Gómez, José E. Zapata, Gabriel López-García, Antonio Cilla, Amparo Alegría

**Affiliations:** 1 Nutrition and Food Technology Group, Faculty of Pharmaceutical and Food Sciences, University of Antioquia, Medellin 050010, Colombia; ljohanna.gomez@udea.edu.co (L.J.G.); natgogri@gmail.com (N.A.G.); edgar.zapata@udea.edu.co (J.E.Z.); 2 Nutrition and Food Science Area, Faculty of Pharmacy, University of Valencia, Avda. Vicente Andrés Estellés s/n, 46100 Burjassot, Valencia, Spain; gabriel.lopez@uv.es (G.L.-G.); amparo.alegria@uv.es (A.A.)

**Keywords:** protein hydrolysis, surface response design, iron-binding activity, iron bioavailability, Alcalase, Caco-2 cells

## Abstract

Iron deficiencies continue to cause significant health problems in vulnerable populations. A good strategy to combat mineral deficiency includes fortification with iron-binding peptides. This research aims to determine the optimal conditions to hydrolyze red tilapia viscera (RTV) using Alcalase 2.4 L and recovery of iron-binding protein hydrolysate. The result showed that under the optimal hydrolysis condition including pH 10, 60 °C, E/S ratio of 0.306 U/g protein, and substrate concentration of 8 g protein/L, the obtained hydrolysate with 42.5% degree of hydrolysis (RTVH-B), displayed the maximal iron-binding capacity of 67.1 ± 1.9%. Peptide fractionation was performed using ultrafiltration and the <1 kDa fraction (FRTVH-V) expressed the highest iron-binding capacity of 95.8 ± 1.5%. Iron content of RTVH-B and its fraction was assessed, whereas iron uptake was measured indirectly as ferritin synthesis in a Caco-2 cell model and the result showed that bioavailability of bound minerals from protein complexes was significantly higher (*p* < 0.05) than iron salt in its free form, increased 4.7 times for the Fe^2+^–RTVH-B complex. This research suggests a potential application of RTVH-B as dietary supplements to improve iron absorption.

## 1. Introduction

The by-products of the fish industry represent between 50% and 70% of the weight of the animal, of which generally only 30% is used, while the remaining 70% is wasted [1]. This situation makes it necessary to search for profitable and environmentally friendly methods to make use of this type of waste. The development of specific hydrolysates or peptides with functional and bioactive properties is one of the alternatives for the use of these by-products, since these peptides have a great potential as value-added ingredients for the pharmaceutical or food industry, since they are considered safe, nutritionally healthy, low cost, and with therapeutic benefits [2].

Iron, as an essential element, participates in many biochemical processes in the human body including oxygen transport, energy production, and cell proliferation. However, nearly one-fifth of the world’s population has been reported to have nutritional problems due to iron deficiency. This is due to its low absorption rate, which is between 15 and 35% in the heme chemical form and below 10% in the non-heme form [3]. For this reason, several studies are now focusing on iron fortification and supplementation with this mineral [4]. Iron salts are the most popular form of supplementation, but are being replaced because of stability problems, low bioavailability, and absorption into the body, due to their reactivity with other food components, such as phytic acid, polyphenols, and certain fibers [5]. An alternative that has gained momentum in the past decade is the use of hydrolysates obtained from proteolytic digestion of different food sources, which have demonstrated considerable capacity to bind to metal ions and improve their stability, solubility, and bioavailability, because of their spatial structure and their various residues with side chains capable of donating electrons [6]. In recent years, chelating peptides obtained from hydrolysates of different protein sources have been reported, including proteins from fish by-products such as collagen from fish scales [5], cod skin [7], akiami paste shrimp Acetes japonicus [8], Nile tilapia skin [9], and red tilapia scales [10].

In most cases, the chelating properties of bioactive peptides have been determined using only chemical methods, but more detailed studies determining binding constants or biological properties through in vitro or in vivo studies are limited [11]. In this sense, the Caco-2 cell line is appropriate for iron bioavailability studies in food because it is a useful model for evaluating iron absorption at the intestinal level [12]. These cells undergo spontaneous differentiation in culture to form a monolayer of polarized epithelial cells that have many characteristics of enterocytes, with rapid replication and a behavior similar to the human intestinal mucosal barrier, which predicts passive absorption conditions in humans [13].

In this study, we evaluated the ability of red tilapia viscera hydrolysates (RTVH) to bind iron. First, we carried out an experimental design to define the operating conditions that allow the degree of hydrolysis (DH) to be maximized, in search of more active low-molecular-weight peptides. We then determined the effect of the DH and the molecular weight distribution on iron chelating activity and evaluated the capacity of the hydrolysate to improve the iron bioavailability in Caco-2 cells.

## 2. Materials and Methods

### 2.1. Reagents

Alcalase 2.4 L was acquired from Novo Nordisk Co. (Bagsvaerd, Denmark). 3-(4,5-dimethylthiazol-2-yl)-2,5-diphenylthiazolium bromide (MTT) was purchased from Sigma-Aldrich (Oakville, ON, Canada). Spectro Ferritin kit, Catalogue number S-22 was purchased from Ramco Laboratories Inc. (Stafford, TX, USA). HEPES, antibiotic solution (penicillin-streptomycin), Dulbecco’s modified eagle medium (DMEM + GlutaMAXTM), phosphate buffered solution (PBS), non-essential amino acids, fetal bovine serum (FBS), and trypsin-EDTA solution (2.5 g/L trypsin and 0.2 g/L EDTA) were acquired from Gibco (Scotland, UK).

### 2.2. Samples

Different production batches of Red tilapia (*Oreochromis* spp.) viscera were obtained of Piscícola el Gaitero (Antioquia-Colombia). Red tilapia viscera (RTV) were homogenized and heated to 90 °C for 20 min to inactivate the endogenous enzymes and remove most of the fat, as previously mentioned [14]. Subsequently, the aqueous fraction was stored at −20 °C until hydrolysis.

### 2.3. Enzymatic Hydrolysis

#### 2.3.1. Determination of Total Peptide Bonds in the Protein (ht)

The concentration of the α-amino groups in the homogenized and defatted RTV was determined using the o-phthaldialdehyde (OPA) method reported by Nielsen, Petersen, and Dambmann [15]. The total content of α-NH was calculated after total hydrolysis of the viscera with HCl 6N at 110 °C for 24 h. Total of 150 µL of the sample, control (distilled water), or standard (serine at 0.9516 meqv/L), was mixed with 950 µL of the OPA reagent and incubated for 2 min, to subsequently measure its absorbance at 340 nm. The number of total peptide bonds in the native protein per unit weight in meqv/g of protein, was calculated using Equation (1) below.

(1) hT=[Am−AbAe−Ab×0.9516×1P]−BA,

where *P* represents the concentration of protein in g/L, *A_m_*, *A_b_*, and *A_e_* are the absorbances of the sample, the blank and the standard, respectively. For *A* and *B* we used the values of 1 and 0.4 reported by Alder-Nissen [16] for this type of substrate, calculated as the intercept and the slope, respectively, of the correlation between the meqv of the α-amino groups formed during hydrolysis and leucine amino equivalents.

#### 2.3.2. Hydrolysis Process

The hydrolysis was carried out in a 500 mL reactor with a substrate concentration (S), enzyme substrate ratio (E/S), pH, and temperature (T) defined in the experimental design (see Section 2.3.3). A combined glass electrode connected to an automatic titrator (Titrando 842, Metrohm) was used to control pH and T. The reaction was monitored through the DH, expressed as the ratio between the number of hydrolyzed peptide bonds (h) and ht. The DH was calculated with Equation (2), using the pH-stat method [17].

(2) $$DH\;\left(\%\right)=\frac{B\;N_{B}}{M_{p}\;\alpha \;h_{t}}\times 100,$$

where B is the consumed volume of the base in L, M_p_ is the mass of the protein in kg, N_B_ is the concentration of the base, and α is the degree of dissociation of the amino groups released during the reaction. α and pK were calculated with Equations (3) and (4), respectively [17].

(3) $$\propto =\frac{10^{\mathrm{pH}-\mathrm{pK}}}{\left(1+10^{\mathrm{pH}-\mathrm{pK}}\right)},$$

(4) $$\mathrm{pK}=7.8+\frac{\left(298-T\right)}{298*T}*2400,$$

#### 2.3.3. Effect of Substrate Concentration, Substrate Enzyme Ratio, pH, and Temperature on RTV Hydrolysis

In order to identify the best operating conditions in the enzymatic hydrolysis of RTV with Alcalase 2.4 L, we proposed a central composite rotatable surface response design because it is an appropriate design for process optimization [18], using the substrate concentration (S) (8–16 g protein/L), enzyme substrate ratio (E/S) (0.153–0.306 U/g protein), pH (8–10), and temperature (T) (50–60 °C) as factors, taking DH as the response variable. We conducted 30 experiments according to the experimental design, as indicated in Table 1. We used the Design-Expert ^®^ 8.0.5 software (Stat-Ease, Minneapolis, MN, USA) to generate and analyze the data of the experimental designs.

Additionally, we optimized the polynomial model obtained to determine the S, E/S, pH, and T conditions that maximize DH, using the response surface methodology. We verified the optimal conditions predicted by performing three experimental replicates and comparing these results with those predicted by the adjusted polynomial model. Finally, we analyzed the iron chelating capacity, at different DH, of the hydrolysates obtained under optimal conditions.

### 2.4. Fractionation of RTV Hydrolysate

The RTVH with a DH which exhibited higher iron-binding activity (RTVH-B) was centrifuged at 7000× *g*, 10 °C for 15 min (U-320 R, Boeco, Hamburg, Germany). Subsequently, ultrafiltration membranes with molecular weight cut-offs of 100, 10, 3, and 1 kDa, in sequential order (Amicon stirred cells and Amicon ultra-15, Millipore, Darmstadt, HE, Germany) was used to fractionate the supernatant. The fractions were designated as follows: FRTVH-I > 100 kDa fraction, FRTVH-II 10–100 kDa fraction, FRTVH-III 3–10 kDa fraction, FRTVH-IV 1–3 kDa fraction, and FRTVH-V <1 kDa fraction.

### 2.5. Iron Determination

The iron content in samples was determined by flame atomic absorption spectrometry (FAAS) as described in NTC-5151 [19]. The sample was dried, incinerated, and subsequently a digestion was carried out in a heating plate with hydrochloric acid. Atomization was performed using air/acetylene flame and an iron hollow cathode lamp was used for absorbance measurements.

### 2.6. In-Vitro Iron-Binding Capacity

Chelation percentage of iron was determined by measuring the formation of the Fe (II)-ferrozine complex according to Zhu, Wang, and Guo [20]. Approximately, 40 μL of ferrozine (5 mM) was added to 20 µL of FeSO_4_ and 1 mL of sample (0.2 mg protein/mL) or distilled water for control. After 10 min incubation at 25 °C, the absorbance of a colored complex generated by Fe (II) ionic binding to ferrozine was measured at 562 nm (GENESYS 10S UV-Vis, Thermo Scientific™, Waltham, MA, USA). Chelation percentage of iron was calculated using Equation (5) below. Where *A*_1_ is the sample absorbance and *A*_0_ is the control absorbance.

(5) $$Chelation\;percentage\;of\;Fe\;\left(\%\right)=\left(1-\frac{A_{1}}{A_{0}}\right)\times 100,$$

### 2.7. Cytotoxic Assays

Human colon adenocarcinoma cells (Caco-2) were purchased from the American Type Culture Collection (ATCC) (HTB-37, Rockville, MD, USA). Cells were maintained and grown in DMEM + GlutaMAXTM and used between passages 15 and 20. Cell were incubated at 37 °C in a 95% of humidified atmosphere with 5% (*v*/*v*) CO_2_, as previously described by López-García, Cilla, Barberá, and Alegría [21]. Caco-2 cells were seeded at a density of 5 × 10^4^ cells/cm^2^ onto 24-well plates (Costar Corp., Greenwich, CT, USA) with 1 mL of DMEM, and the culture medium was changed every two days.

Measuring the transepithelial electrical resistance (TEER) was used to confirm cell differentiation, which reached its maximum value at 7 days post-seeding (722 ± 88.7 Ω cm^2^) and remained constant until 12 days (data not shown). Seven days post-seeding, to quantify cell viability (MTT test), RTVH-B and FRTVH-V were used at different concentrations (0.05–0.5 mg protein/mL), concentrations that are within the range of magnitudes reported by other authors [22,23]. Culture medium was aspirated from the wells and cell cultures were preincubated for 24 h with RTVH-B and FRTVH-V in DMEM FeF. Following that, the culture medium was removed, and the cells were washed with PBS at 37 °C.

Cell viability was determined indirectly using a colorimetric method based on the reduction of the tetrazolium ring of MTT via mitochondrial dehydrogenases (MTT assay) [24]. After exposing Caco-2 cells to RTVH-B and FRTVH-V at different concentrations for 24 h, 0.5 mg/mL of MTT in PBS was added, and the plates were incubated at 37 °C for 2 h. After the MTT solution was removed, insoluble formazan was dissolved in 2-propanol acid, and the optical density was measured at 570 nm, with background subtraction at 690 nm (Perkin Elmer Lambda 2 UV/Vis, Spectrometer, Waltham, MA, USA). The percentage of cell viability was calculated using untreated cells as a positive control (100% cell viability). All experiments were carried out with four replicates.

### 2.8. Iron Bioavailability

#### 2.8.1. Cell Culture

Caco-2 cells were maintained and grown according to the methodology described in Section 2.7. The cells were seeded at a density of 50,000 cells/cm^2^ in 6-well plates and medium was changed every 2 days. To avoid interference from iron sources other than the samples, 5 days post-seeding the cells were maintained under low iron conditions according to Caetano et al. [13]. To prepare the fetal bovine serum (FBS) depleted of iron (DMEM FeF) the following methodology is performed: The pH of FBS was adjusted to 4.5 and mixed with 300 g/L Chelex for 2 h; then the pH was readjusted to 7.2 and FBS was incubated at 25 °C overnight, passed through a common filter paper, and sterilized through a 0.22 μm filter. Cells were maintained in DMEM FeF. The iron uptake assays were carried out 7 days post-seeding, with differentiated cells previously characterized by the transepithelial electrical resistance values (>500 Ω cm^−2^). Each experiment was conducted with four replicates.

#### 2.8.2. Ferritin Assay

Immediately before the addition of the RTVH-B and FRTVH-V, the cell growth medium was removed and the cell monolayer was washed with PBS at 37 °C. An aliquot (2 mL) of the RTVH-B and FRTVH-V to non-cytotoxic concentrations with/without FeSO_4_ (final concentration of 200 µM Fe and 0.45 mg protein/mL), DMEM FeF (blank) or FeSO_4_ (200 µM Fe) was added to the cell monolayer. The cells were incubated for 2 h at 37 °C, 5% CO_2_ and 95% relative humidity. The samples then were replaced with DMEM FeF and returned to the incubator for an additional 22 h.

Ferritin and total protein were determined according to García-Nebot et al. [25]. PBS and trypsin–EDTA solution were used to wash and to detach the cell monolayers, respectively. Subsequently, the cells were collected with 2 mL of cell-culture-grade water and homogenized for 1 min at 4 °C at 17,000 rpm (Polytron PT 2000, Kinematica AG, Lucerne- Switzerland). Ferritin was measured in the cell suspensions by an enzyme immunoassay using the Spectro Ferritin kit. The cell protein content was determined according to Lowry et al. [26]. The ferritin/protein ratio (ng ferritin/mg protein) was used as an index of iron uptake. Control cells (blank) were used throughout the experiments.

### 2.9. Statistical Analysis

The values are expressed as a mean ± standard deviation from at least four separate experiments (*n* = 4). Differences between means were identified by a one-way analysis of variance (ANOVA), followed by the Tukey’s HSD test. The level of statistical significance was set at *p* < 0.05 (Stagraphics^®^ Centurion XV statistical software, The Plain, VA, USA). We used the Design-Expert^®^ 8.0.5 software (Stat-Ease, Minneapolis, MN, USA) to generate and analyze the data of the experimental designs. We tested the adequacy of the model, the statistical significance of the regression coefficients, and the interaction between the different independent variables by means of an ANOVA. We tested the significance of the coefficients estimated in the model with the F statistic (*p*-value) at a 95% confidence level. We assessed the validity of the results obtained in the statistical model according to the assumptions of homogeneity of variances, independence of errors, and normality [18].

## 3. Results

### 3.1. Effect of S, E/S, pH, and Temperature on RTV Hydrolysis

Table 1 presents the randomized experimental runs, with their respective response variable (DH).

The ANOVA (Table 2) shows that the model obtained adequately represents the experimental data (*p* < 0.0001) and has a non-significant lack of fit (*p* > 0.5624). To develop the model, we adjusted the data to a 2-factor interaction model (2FI). The independent variables E/S and pH showed the greatest effect on DH, followed by the S-E/S, E/S-pH interactions, the S variable, and finally the pH-T interaction, respectively. The other interactions and the independent variable T did not show statistical significance on the response (*p* > 0.05).

Equation (6) presents the polynomial describing the influence of the independent variables S, E/S, pH, and T, with their interactions, on RTV protein DH, which can be considered reliable since ANOVA meets the assumptions of normality, constant variance, and independence [18].

**Figure 1:**
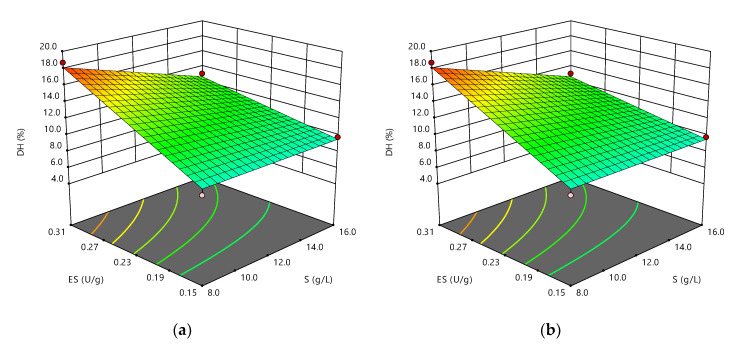

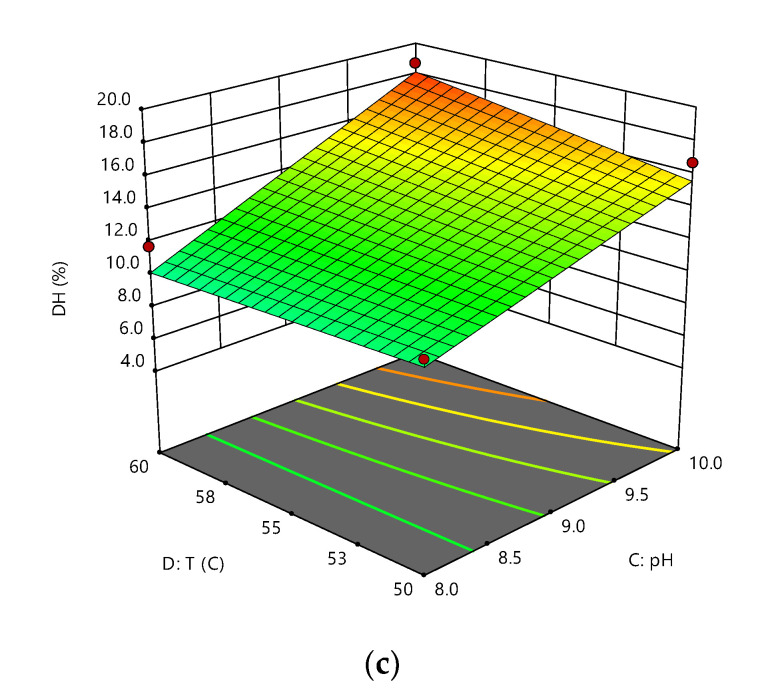
Response surface plots of the relative effects on the DH of (**a**) S and E/S; (**b**) E/S and pH; (**c**) pH and T.

(6) GH=79.26+(0.79×S)−(52.10×E/S)−(9.5×pH)−(1.32×T)−(4.36×S×E/S)+(14.15×E/S×pH)+(0.15×pH×T),

Figure 1 shows the response surfaces that illustrate the main and interactive effects of the independent factors on DH. To illustrate the interactions between variables, which presented statistical significance (*p* < 0.05), Figure 1a shows the effect of S and E/S keeping pH and T fixed at their optimum values, Figure 1b shows the effect of E/S and pH keeping S and T fixed at their optimum values, and Figure 1c shows the effect of pH and T keeping S and E/S fixed at their optimum values.

All figures denote the absence of the quadratic effect of the factors since there are no curves in the surfaces. This implies that the values that maximize the response are at the extremes of the working range. Figure 1a shows that the highest DH values are obtained at low substrate concentrations, when working at the upper limit of E/S, which has an adverse technological effect since the energy consumption for obtaining a certain amount of dry hydrolysate is increased [27]. Further, response surface plots show that increases in pH have a positive effect on DH in the study range, mainly at high temperatures (Figure 1c) and high E/S ratios (Figure 1b).

### 3.2. Effect of DH on Iron-Binding Activity of Hydrolysates

**Figure 2:**
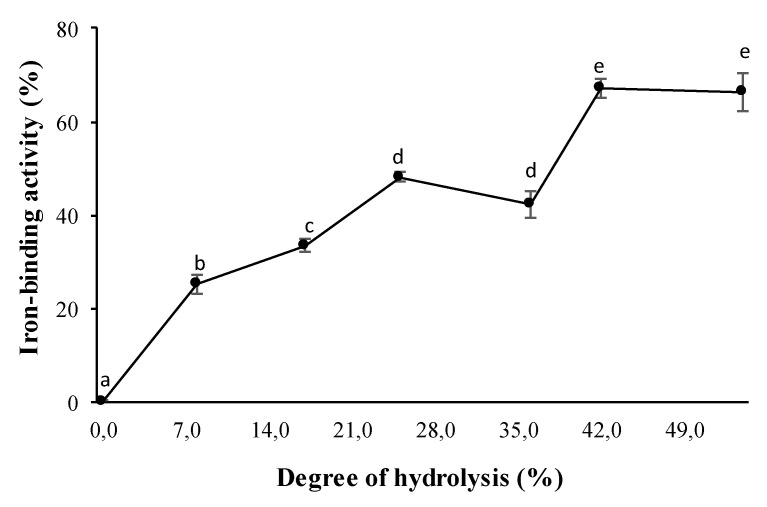
Iron-binding activity of RTVH-B with different DHs. Values are expressed as means ± standard deviation (*n* = 4). Different superscript letters (a–e) denote statistically significant differences among treatments (*p* < 0.05).

We evaluated the iron-binding capacity of the hydrolysates obtained under the conditions defined in the optimization process as a function of the DH (Figure 2). The chelating capacity increases proportionally with DH, reaching a maximum value of 67.1 ± 1.9% with the hydrolysate with DH of 42.6% (RTVH-B), after which increases in DH do not significantly favor the activity (*p* > 0.05). So the results suggest that the process of enzymatic hydrolysis of proteins is an efficient process for obtaining iron-binding peptides and increases in DH lead to the production of bioactive hydrolysates with higher iron chelating activity.

### 3.3. Fractionation by Molecular Weight of the Hydrolysate with the Highest Iron Chelating Activity

**Figure 3:**
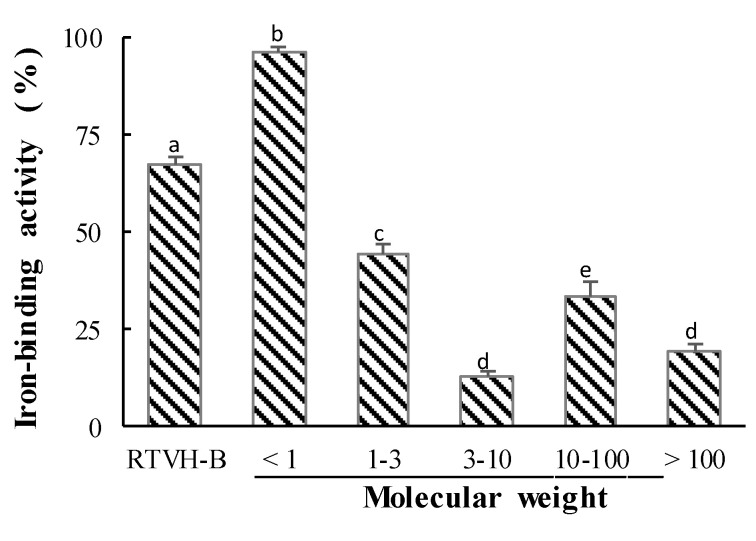
Iron-binding activity of RTVH-B and its molecular weight fractions. Values are expressed as means ± standard deviation (*n* = 4). Different superscript letters (a–e) denote statistically significant differences among treatments (*p* < 0.05).

We fractionated the RTVH-B, which presented the highest iron chelating activity, and the results can be seen in Figure 3. It shows that the FRTVH-V fraction (<1 kDa, 95.8 ± 1.5%) has 28.7% more activity than that of the complete hydrolysate, while the fractions with higher molecular weight show significantly lower activity than that found in the complete hydrolysate (*p* < 0.05). These results indicate that the molecular mass of RTVH is an important factor in iron-binding capacity and that low-molecular-weight peptides favor such activity.

**Figure 4:**
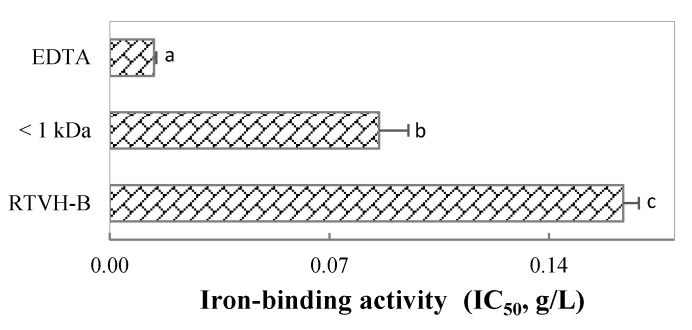
IC_50_ of RTVH-B, its fraction FRTVH-V, and EDTA. Different superscript letters denote statistically significant differences among treatments (*p* < 0.05).

To compare the activity obtained with that of a commercial chelator, we determined the iron chelating capacity of RTVH-B, its FRTVH-V fraction and EDTA (ethylenediaminetetraacetic acid, chelating agent), at different concentrations and calculated IC_50_, defined as the concentration necessary to chelate 50% of the total iron. The results are shown in Figure 4, where it can be seen that the IC_50_ of the hydrolysate is almost double that of the fraction, which implies that the most active peptides of the hydrolysate have a molecular weight of less than 1 kDa and that they have twice the activity of the complete hydrolysate.

EDTA is one of the most important synthetic chelating agents in the industry, because of its high capacity to form coordinated complexes with most multivalent metal ions [28]. Therefore, the fact that the IC_50_ of the FRTVH-V fraction is in the same order of magnitude as EDTA implies that this fraction has high potential for application in the industry.

### 3.4. Bioavailability of Iron

#### 3.4.1. Cytotoxicity Analysis

To define the non-toxic concentrations of RTVH-B and FRTVH-V, which showed the highest iron-binding activity, we performed a cytotoxicity analysis using the MTT test. For this, we treated differentiated Caco-2 cells with different concentrations of RTVH-B and FRTVH-V (0.05–0.5 mg/mL). These concentrations were chosen based on previous studies where the effect of hydrolyzed fish by-products on human cells was evaluated [22,23].

**Figure 5:**
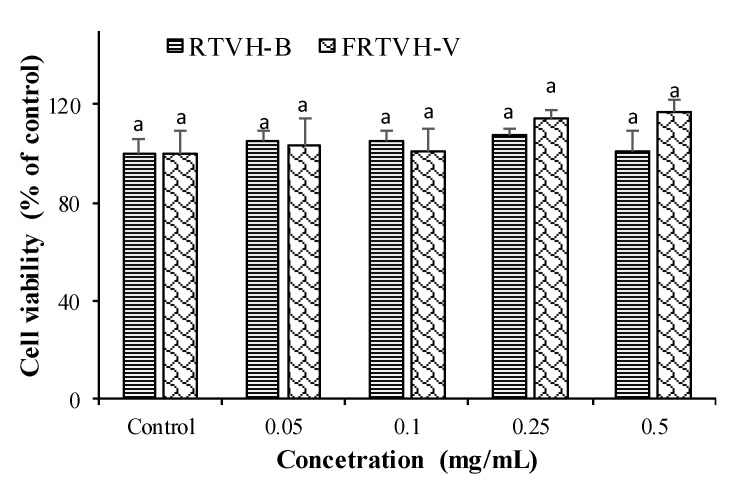
Effect of RTHV-B and FRTVH-V on cell viability (MTT assay) in differentiated Caco-2 cells after 24 h treatment. Values are expressed as means ± standard deviation (*n* = 4). Different superscript letters denote statistically significant differences among treatments (*p* < 0.05).

Figure 5 shows that the samples do not produce significant effects on the mitochondrial function of the cells after 24h of treatment, at any of the concentrations evaluated (*p* > 0.05), so it is safe to use RTVH-B and the fraction in this concentration range to evaluate the iron chelating effect.

#### 3.4.2. Effect of RTVH-B and FRTVH-V on In Vitro Iron Bioavailability

Currently, iron-peptide complexes are considered a promising source of more bioavailable, stable iron with reduced side effects compared to iron salts [29]. For this reason, we studied the effect of the addition of RTVH-B and FRTVH-V on intestinal iron uptake using Caco-2 cells and an iron concentration of 200 µM. As the Caco-2 cells are able to synthesize ferritin in response to increased intracellular iron level [30], the ferritin formation in the cells was used as an indicator of iron bioavailability. We incubated Caco-2 cells as described in the methodology with RTVH-B and FRTVH-V with/without FeSO_4_, FeSO_4_ only, and culture medium as cell control. For the analysis, we used RTVH-B and FRTVH-V at a concentration of 0.45 mg of protein/mL, which was shown to be safe for Caco-2 cells.

**Figure 6:**
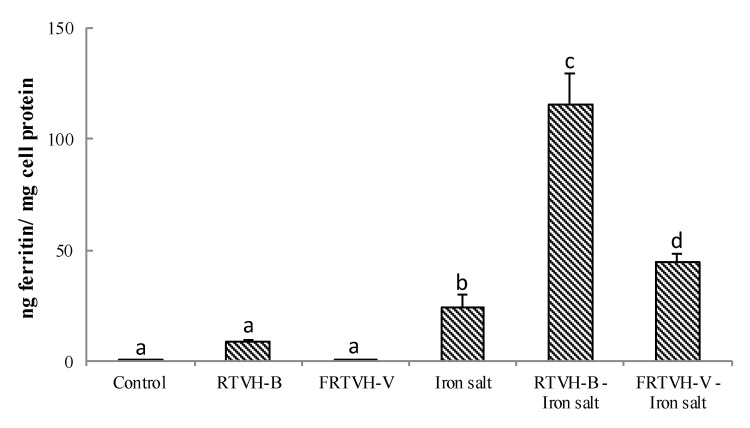
Iron bioavailability (ng ferritin/mg cell protein) of cells treated with iron salt (FeSO_4_·7H_2_O) in free and complexed forms. Control: cells treated with DMEM. Values are expressed as means ± standard deviation (*n* = 4). Different superscript letters (a–d) denote statistically significant differences among treatments (*p* < 0.05).

Iron uptake expressed as a ratio of ferritin and cell protein (ng ferritin/mg cell protein) is shown in Figure 6. Initial iron content found by FAAS in RTVH-B was 5.17 µg Fe/mg protein, while in the FRTVH-V the value found was below the detection limits (<1 µg Fe/mg protein). For this reason, in cells treated only with RTVH-B the ferritin content increases non-significantly from 0.9 ± 0.1 ng ferritin/mg protein for the control to 8.9 ± 0.6 ng ferritin/mg protein, that is, the value was nine-fold greater than control.

Both treatments, RTVH-B-FeSO_4_ and FRTVH-V-FeSO_4_, significantly increase the bioavailability of iron with respect to the control (*p* < 0.05). As shown in Figure 6, the concentration of ferritin in Caco-2 cells increased from 24.3 ± 5.1 to 115.7 ± 13.4 ng ferritin/mg cell protein because of the addition of RTVH-B (4.7 times) and up to 44.7 ± 3.6 ng ferritin/mg protein with FRTVH-V (1.8 times).

## 4. Discussion

The dependence of DH on E/S has been widely reported [31,32] and is common when enzyme concentrations are relatively low in relation to critical saturation-inducing concentrations in enzyme hydrolysis reaction systems [33]. Figure 1a shows that the highest DH values are obtained at low substrate concentrations. But it is a fact that increases in substrate concentration generally lead to decreases in final DH and hydrolysis rate [31,34], as has been reported in the hydrolysis of bovine hemoglobin [35], α-lactalbumin [27], and trout viscera [36]. It was initially attempted to explain this behavior on the basis that the high viscosity of the solvent when increasing the substrate concentration may result in a lower reaction speed [37]. However, this hypothesis has been ruled out [38], as well as oligomerization or the formation of insoluble substrate aggregates [39]. It has been suggested that there may be changes in the quality of the solvent or modifications in the structural stability of the substrate or enzyme, which affect the accessibility of the enzyme to the substrate [39]. It has also been proposed that there are alterations in the relative speed at which each cleavage site is hydrolyzed, i.e., in the selectivity of the enzyme. Arguing that low concentrations of substrate deliver less intact protein than high concentrations, for the same DH [38]. More recently, the formation of enzyme inhibitory peptides in the same hydrolysis reaction has been hypothesized [27].

Response surface plots show that increases in pH have a positive effect on DH, as has been reported in tilapia skeleton protein [40] and in other studies that state that alcalase shows efficient catalytic activity at alkaline pH and remains active up to pH 6 and a temperature range between 55 and 70 °C, the optimal value being dependent on the substrate used [41]. However, very high pH values can denature proteins and damage some amino acids [42]. In general, the effect of pH on protein hydrolysis is based on the fact that this variable affects both the substrate and the enzyme, because it changes the distribution of loads and the conformation of the proteins [43] and modifies the enzyme–substrate association dynamics, because it can influence the dissociation of the enzyme’s active groups [35].

For the working range of the present study, no statistically significant effects of temperature on DH were observed, but a significant interaction with pH (*p* value = 0.0226) is presented. These two variables are of great interest in enzymatic reactions, given that, because of their protein nature, extreme values of these factors can lead to denaturation and loss of the enzyme’s catalytic activity. For this reason, it is necessary to define the best conditions for the substrate in each case. For example it has been possible to maximize the DH in terms of pH and temperature of bovine plasma at pH 8.32 and 54.1 °C [44] and pH 9 and 61.5 °C [45], tilapia by-products at pH 7.5 and 60 °C [46], channeled applesnail at pH 10 and 45 °C [47], trout viscera at pH 8.5 and 60 °C [36].

The importance of DH lies in the fact that its increase has been associated with a greater presence of low-molecular-weight peptides, which in turn are attributed to greater biological activity [48,49]. In this sense, it has been reported that the molecular weight of hydrolysates is an important factor in iron chelating activity and that in general, low-molecular-weight peptides, between 1 and 5 kDa, show the highest activity [50,51]. Therefore, obtaining hydrolysates with high DH improves the probability that the peptides obtained present biological activity of some kind [52]. For these reasons, we subjected the model obtained (Equation (6)) to an optimization procedure with the response surface methodology, in order to predict the levels of the factors that maximize DH in the three hours of reaction. The optimal conditions for the RTV enzymatic hydrolysis reaction with Alcalase 2.4 L were: S 8 g/L, E/S 0.306 U/g, pH 10, and T 60 °C. The predicted value for DH under optimal conditions was 17.5% and that obtained experimentally was 16.43 ± 0.57%. Thus, with a relative error of 6.1%, it can be said that the optimization process is valid to maximize DH under the working conditions of this study.

We evaluated the iron-binding capacity of RTVH with different DH. It can be observed that the hydrolysis process increases the ability of RTV to bind iron, which has already been reported [53,54]. DH above 42.6% does not significantly favor activity, in this regard, Wu, Li, Hou, Zhang, and Zhao [7] reported that the iron chelating capacity of gelatin hydrolysates from cod skin increases with increasing DH and reaches a maximum value of 17.5 ± 0.3%. Similarly, Guo et al. [3] found that the iron chelating activity of Alaskan pollock skin hydrolysates increases rapidly from 17 to 28% over a hydrolysis time of 15 to 120 min, after which the activity increased slightly to a maximum of ~35% at 360 min reaction time. Torres-Fuentes, Alaiz, and Vioque [55] reported an iron chelating capacity of ~15% for chickpea protein hydrolysates, obtained by sequential action of the digestive enzymes pepsin and pancreatin. We can note that the iron chelating activity found in the RTVH-B in the present study is superior to those previously reported. This suggests that RTV are a good source of iron chelating peptides and that hydrolysis with Alcalase 2.4 L increases the iron binding sites because of the multiple peptides released during the process [29]. The correlation between DH and chelating activity is due to the decrease and change in the molecular structure of the peptides released, to the exposure of an increasing number of negatively charged motifs, such as carboxyl groups (-COO-), which may be the iron-binding sites [51] and to the decrease of steric effect caused by the large size of the proteins [56].

The size of peptides can play an important role in their ability to chelate metal ions. The chelating activity of peptides depends on molecular weight, peptide structure, amino acid composition, and steric effects [3]. We found that small peptides of less than 1 kDa show the highest iron chelating activity. In this sense Sun et al. [51] found a positive correlation (*r* = 0.88, *p* < 0.05) between the proportion of small fractions (between 0.2 and 1 kDa) and iron-binding activity. In the same way, Vo et al. [8] indicated that low-molecular-weight hydrolysates (1–3 kDa), derived from Acetes japonicus, have better iron-binding activity than high-molecular-weight hydrolysates.

The high iron-binding capacity presented by low-molecular-weight peptides may be due to an increased exposure of the side chains of the amino acid residues, which facilitates the interaction between an electron donor group on the surface of the peptide and the metal ion [50]. It has been reported that there is an excellent linear relationship between His-containing peptides and their ability to chelate metals, which has been related to the imidazole ring of the His residue, especially when His is at the C-terminal end, which can chelate the metal ion through a coordination bond [57]. On the other hand, Cruz-Huerta et al. [58] reported that in whey protein the iron chelating peptides mainly contain Asp, Glu, and Pro amino acids. Additionally, some amino acid side chain groups such as the amino from Lys and Arg, and carboxyl from Asp, also played a crucial role in the formation of iron-peptide complexes [7]. So considering the relationship of peptide structure and amino acid composition over iron-binding peptides activity. It is important in future investigations to use mass spectrometric methods to elucidate the peptide sequence of the active peptides in both samples RTVH-B and FRTVH-V.

We find that concentrations of RTVH-B and FRTVH-V between 0.05 and 0.5 mg/mL do not produce cytotoxicity in the Caco-2 cells after 24 h of treatment. Similarly, other authors have found that hydrolysates of tilapia by-products are not cytotoxic on some cell lines. In this context, Ngo et al. [22] found that concentrations less than 1 mg/mL of Nile tilapia (Oreochromis niloticus) scale gelatin hydrolysates are not cytotoxic for mouse macrophages and human lung fibroblasts cell lines. On the other hand, peptides have become increasingly important and influential for the treatment of many human diseases and compared to traditional small molecule drugs, peptides have better safety profiles low toxicity [2].

The results showed that the addition RTVH-B-FeSO_4_ and FRTVH-V-FeSO_4_ complexes, significantly increase the bioavailability of iron with respect to iron salt in its free form. This increase in iron uptake by cells in the presence of RTVH-B and FRTVH-V compared to the use of the iron salt alone is probably due to the fact that under physiological conditions ferrous iron (Fe^2+^) is rapidly oxidized to the ferric (Fe^3+^) form [11], which is easily hydrolyzed to form iron hydroxide Fe(OH)_3_ that is insoluble in aqueous solutions at neutral pH [59]. The chelating peptides present in RTVH-B and FRTVH-V can protect iron from binding to water, which hinders the formation of iron hydroxides and increases iron solubility, resulting in increased availability of iron for absorption [29]. Similar findings have been reported by Eckert et al. [11], who found that the ferritin content in Caco-2 cells increases three times when using the peptide SVNVPLY to chelate the iron, going from 40.8 ng ferritin/mg protein when cells are incubated with FeSO_4_ to 122.8 ng ferritin/mg protein when using the complex Fe^2+^-SVNVPLY. Caetano et al. [13] determined that a fraction of less than 5 kDa of whey protein hydrolysate increases iron absorption in vitro in models with Caco-2 by 1.3 times compared to the iron salt FeCl_2_. Similarly, some fractions obtained by in vitro digestion of cow’s milk increase the amount of ferritin synthesised in Caco-2 cells by up to 3.5 times compared to the control [60].

Figure 6 also shows that treatment with RTVH-B-FeSO_4_ led to a higher accumulation of ferritin than that presented with FRTVH-V-FeSO_4_, although the affinity of bonding to iron in the chemical tests carried out previously appeared to be higher for FRTVH-V than for the complete hydrolysate (Figure 4). These results suggest that the increase in iron bioavailability does not directly correspond to the chelating activity of the metal found by chemical methods, which shows the importance of reinforcing these analyses with the study of the biological properties of the complexes formed, in terms of absorption and transport in the digestive system, either by in vitro methods with cell lines or in vivo methods. The increased activity of RTVH-B may be due to the fact that as charged entities, free metal ions require ionophore mediated transport to cross the lipid bilayer of cells. For this reason, a chelator that effectively improves the bioavailability of metal ions must not only have the capacity to bind to iron and increase its solubility at physiological pH, but also present a high rate of dissociation of the chelate–metal complex, because the transport process can be considered mainly as an exchange reaction of ligands between the chelator and the transporter [59]. Thus the high capacity of RTVH-B to increase the bioavailability of iron compared to FRTVH-V may suggest that RTVH-B-Fe^2+^ complexes have a lower bond strength than FRTVH-V-Fe^2+^ complexes, and thus allow a higher rate of ligand exchange leading to a higher availability of iron to bind to transporters and be uptaken by the cell.

Ferrous sulfate is one of the most common compounds used for food fortification for anemia control [13]. Hence, in comparing the bioavailability of FeSO_4_, RTVH-B-Fe^2+^ complex is a potential alternative for food fortification because its bioavailability was approximately four-fold higher than that of this reference salt.

## 5. Conclusions

The results showed that S, E/S, and pH have significant effect on the efficiency of the RTV hydrolysis reaction with Alcalase 2.4 L, with E/S and pH being the most influential factors. We also demonstrated that the operating conditions that maximize the DH of the RTV protein-Alcalase 2.4 L reaction system are: S 8 g protein/L, pH 10, temperature 60 °C, and E/S 0.306 U/g protein.

On the other hand, we found that the process of enzymatic hydrolysis of proteins is an efficient process for obtaining iron-binding peptides, and we found that increases in DH lead to the production of bioactive hydrolysates with higher iron chelating activity, reaching the highest activity with RTVH-B (DH: 42.6%). In terms of bioavailability testing we identified that iron uptake from RTVH-B-Fe^2+^ complex was four-fold greater than treatment with iron salt. All these results indicate that enzymatic hydrolysis is a suitable strategy for the exploitation of RTV, as it is possible to obtain iron-binding peptides that could be used as supplements for the treatment of iron deficiency disorders.

## Figures and Tables

**Table 1:** Central composite rotatable surface response design for evaluation of the substrate concentration (S), enzyme substrate ratio (E/S), pH and temperature (T) S, E/S, pH, and T effect on the degree of hydrolysis degree of hydrolysis (DH) of red tilapia viscera (RTV) with Alcalase 2.4 L.

Run	S (g/L)	E/S (U/g)	pH	T (°C)	DH (%)
1	12	0.23	9	55	9.73
2	16	0.15	10	60	9.78
3	8	0.23	10	50	8.23
4	12	0.23	9	55	11.48
5	12	0.23	9	55	9.48
6	12	0.23	9	65	9.81
7	12	0.08	9	55	6.34
8	16	0.31	8	50	8.94
9	12	0.23	9	55	7.69
10	16	0.15	8	50	8.88
11	8	0.15	10	60	8.95
12	8	0.31	10	60	18.68
13	8	0.15	8	50	7.21
14	12	0.23	9	45	8.33
15	16	0.31	10	60	13.06
16	12	0.38	9	55	10.74
17	4	0.23	9	55	9.41
18	12	0.23	9	55	8.99
19	20	0.23	9	55	7.63
20	16	0.31	10	50	11.80
21	12	0.23	9	55	9.61
22	8	0.31	8	60	11.78
23	16	0.15	10	50	7.90
24	8	0.31	8	50	11.07
25	16	0.31	8	60	5.28
26	16	0.15	8	60	6.48
27	12	0.23	7	55	5.89
28	8	0.31	10	50	16.69
29	8	0.15	8	60	6.47
30	12	0.23	11	55	14.76

**Table 2:** Analysis of variance of the experimental design to evaluate the effect of substrate concentration (S), enzyme substrate ratio (E/S), pH, and temperature (T), on the hydrolysis of RTV.

Source Model	F Value	*p*-Value
Model	23.106	<0.0001
A-S	11.896	0.0023
B-ES	50.195	<0.0001
C-pH	61.519	<0.0001
D-T	0.209	0.6523
AB	19.224	0.0002
BC	12.682	0.0017
CD	6.015	0.0226
Lack of Fit	0.982	0.5624
R^2^	0.880	
Adjusted R^2^	0.842	
